# Intentional wedge resection versus segmentectomy for ≤2 cm ground-glass-opacity-dominant non-small cell lung cancer: a real-world study using inverse probability of treatment weighting

**DOI:** 10.1097/JS9.0000000000001361

**Published:** 2024-03-21

**Authors:** Chengwu Liu, Zhenyu Yang, Yiming Li, Chenglin Guo, Liang Xia, Weiheng Zhang, Congjia Xiao, Jiandong Mei, Hu Liao, Yunke Zhu, Feng Lin, Lin Ma, Qiang Pu, Lunxu Liu

**Affiliations:** a Department of Thoracic Surgery, West China Hospital; b Western China Collaborative Innovation Center for Early Diagnosis and Multidisciplinary Therapy of Lung Cancer, Sichuan University, Chengdu, People’s Republic of China

**Keywords:** ground glass opacity, non-small-cell lung cancer, segmentectomy, survival, wedge resection

## Abstract

**Background:**

Whether wedge resection is oncological suitable for ground glass opacity (GGO)-dominant non-small cell lung cancer (NSCLC) ≤2 cm is still debatable. The aim of this study is to investigate the short-term and long-term outcomes of intentional wedge resection and segmentectomy for those patients.

**Materials and Methods:**

This was a real-world study from one of the largest thoracic surgery centers in West China. Patients who underwent intentional wedge resection or segmentectomy for ≤2 cm CTR (consolidation-to-tumor) ≤0.5 NSCLC were consecutively included between December 2009 and December 2018. Data were prospectively collected and retrospectively reviewed. Inverse probability of treatment weighting (IPTW) was used to balance baseline characteristics. Long-term outcomes, including overall survival (OS), recurrence-free survival (RFS), and lung cancer-specific survival (LCSS), were analyzed using Cox proportional model.

**Results:**

A total of 1209 patients were included (497 in the wedge resection group, 712 in the segmentectomy group). Compared to segmentectomy, wedge resection had a significantly lower rate of complications (3.8 vs. 7.7%, *P*=0.008), a shorter operating time (65 min vs. 114 min, *P*<0.001), and a shorter postoperative stay (3 days vs. 4 days, *P*<0.001). The median follow-up was 70.1 months. The multivariate Cox model indicated that wedge resection had survival outcomes that were similar to segmentectomy in terms of 5-year OS (98.8 vs. 99.6%, HR=1.98, 95% CI: 0.59–6.68, *P*=0.270), 5-year RFS (98.8 vs. 99.5%, HR=1.88, 95% CI: 0.56–6.31, *P*=0.307) and 5-year LCSS (99.9 vs. 99.6%, HR=1.76, 95% CI: 0.24–13.15, *P*=0.581).

**Conclusion:**

Intentional wedge resection is an appropriate choice for ≤2 cm GGO-dominant NSCLC.

## Introduction

Highlights

- Wedge resection had better short-term outcomes for ≤2 cm GGO-dominant NSCLC.
- Wedge resection had equivalent long-term survival outcomes for ≤2 cm GGO-dominant NSCLC.
- Wedge resection is also suitable for 0.25 <CTR ≤0.5 subgroup patients.

One of the main causes of cancer-related deaths is lung cancer, which accounts for ~18 million cancer fatalities annually across the globe and has a low 5-year overall survival rate of less than 20%^1,2^. In 2011, the National Lung Screening Trial found that screening with low-dose computed tomography (CT) reduced the risk of lung cancer mortality^3^. Since then, CT has been frequently utilized, thereby making it possible to detect an increasing number of small-sized lung tumors, particularly nonsolid tumors with ground-glass opacities (GGOs)^4^. Although lobectomy has been the ‘gold-standard’ treatment for early-stage lung cancer since 1995^5^, growing evidence suggests that sublobar resection can offer equal long-term outcomes and preserve lung function in selected patients^6,7^. Therefore, sublobar resection has been widely performed in recent years. Sublobar resection consists of either segmentectomy or wedge resection, and the surgical intensity of these two procedures differs considerably. Wedge resection refers to nonanatomical resection without manipulation of the vessels or bronchi, but can adequately achieve negative surgical margins and preserve a similar portion or even more of the pulmonary parenchyma. Thus, surgery for wedge resection is less complicated, takes less time, and has fewer perioperative complications. However, there is ongoing debate regarding the specific demographics for which wedge resection or segmentectomy in sublobar resection should be performed.

It has been reported that preoperative radiological findings of GGO can accurately predict prognosis. The connection between radiological abnormalities and prognosis in early-stage non-small cell lung cancer (NSCLC) was explored in an observational study (JCOG0201) by the Japan Clinical Oncology Group (JCOG)^8,9^. According to this study, the consolidation-to-tumor ratio (CTR) was a reliable indicator of the invasiveness and prognosis of subsolid lung cancer. Moreover, JCOG conducted several studies to determine the best surgical strategy for treating tumors with various CTRs and of various sizes. The randomized control trial (RCT) JCOG0802/WJOG4607L demonstrates that segmentectomy outperforms lobectomy in terms of overall survival in patients with early-stage lung cancer with a tumor size ≤2 cm and a CTR > 0.5^10^. A nonrandomized phase III trial (JCOG0804/WJOG4507L) indicated that patients with radiologically noninvasive lung cancer who have a tumor size less than 2 cm and a CTR of less than 0.25 are suitable for sublobar resection with no risk of local recurrence^11^. In another clinical trial (JCOG1211), segmentectomy provided good survival outcomes for patients with a tumor size <3 cm and dominant (CTR≤0.5) GGO NSCLC, including GGO tumors exceeding 2 cm^12^. Although many studies have confirmed that the survival outcomes of sublobar resection and lobectomy are comparable for GGO patients^7,13–15^, the optimal surgical procedure (segmentectomy or wedge resection) remains unclear^16–18^. Through retrospective analysis of the clinical data of dominant GGO patients with tumor sizes less than 2 cm who underwent intentional wedge resection or segmentectomy in our department, we aimed to investigate the long-term and short-term outcomes between the two surgical approaches.

## Material and methods

This retrospective study was conducted using the prospectively maintained West China database, which included clinical data of all lung cancer patients who underwent surgery in the Department of Thoracic Surgery, West China Hospital, Sichuan University. All data was automatically retrieved from our hospital records. This study was approved by the Institutional Review Board (IRB) of West China Hospital (no. 2024-56). Informed consent was waived for this research. This study was registered in the XXX. We present the following article in accordance with the strengthening the reporting of cohort, cross-sectional, and case–control studies in surgery (STROCSS) reporting checklist^19^ (Supplemental Digital Content 1, http://links.lww.com/JS9/C204).

### Patients

Small-sized (≤2 cm) GGO-dominant (CTR ≤0.5) patients who underwent intentional segmentectomy or wedge resection were consecutively included between December 2009 and December 2018 in our department. CT images were reviewed by two surgeons and CTR was calculated following the criteria from the Fleischner Society. The comorbidities of patients were scored according to the Charlson Comorbidity Index (CCI). Patients with multiple primary lung nodules were also included. The exclusion criteria were as follows: (1) patients with a previous cancer history; (2) patients with a CTR >0.5; and (3) patients with missing follow-up data. Pathological TNM (pTNM) stage was manually determined according to the eighth edition NSCLC staging system proposed by the IASLC.

### Operative procedure

Preoperative planning was based on high-resolution computed tomography (HRCT) of the chest. By carefully assessing the HRCT, we identified the lung segmental vessels, segmental bronchus, and their adjacent structures; if necessary, 3D reconstruction was used to assist in the identification. Anesthesia, incision, and surgical approaches were conducted as described in previous reports^20^. Nodules were located via intraoperative digital palpation, preoperative CT-guided puncture or a noninvasive 3D printed emulation model^21^. Segmentectomy was conducted through a single-direction strategy. We used the stem-branch method to track the anatomy during segmentectomy^22^. Inflation—deflation, infrared-fluorescence-enhanced and lung surface intersegmental landmarks methods^23^ were used to identify the intersegmental planes, and stapler-based tailoring was used for division. The surgical margins included at least 2 cm (or the maximum diameter of the tumor) margin of normal lung parenchyma. Systematic or lob-specific mediastinal lymph node dissection (MLND) combined with hilar lymph node dissection was routinely conducted. The drainage tube was removed when the chest radiography showed a well-inflated lung, the 24 h chest drainage was ≤300 ml and there was no air leakage^24^. All patients signed informed consent forms before surgery.

### Follow-up

Patients visited the outpatient department 1 month after surgery for postoperative assessment. Then, patients were evaluated every 3 to 6 months during the first 2 years, every 6 months in the next 3 years, and annually thereafter. Chest and abdominal CT scans and brain CT or MRI scans were performed to detect local recurrence or metastases. However, patients who could not visit our hospital were followed-up via telephone call every 6 months. The last follow-up time was September 2023.

### Outcomes

Postoperative complications include persistent pulmonary air leak (>5 days), pneumonia, pulmonary embolism (PE), chylothorax, poor wound healing/infection, etc. Operating time and blood loss were extracted from surgery records. Overall survival (OS) was defined as the period from surgery to death or last follow-up. Recurrence-free survival (RFS) was defined as the period from when the patient underwent surgery to recurrence, metastasis, death, or last follow-up. Lung cancer-specific survival (LCSS) was defined as the period from surgery to death caused by lung cancer or the last follow-up.

### Statistical analysis

R version 4.3.0 (R Foundation for Statistical Computing) was used for statistical analysis. A *χ*^2^ test was used to compare categorical variables, and Fisher’s exact test was used when some of the groups had small counts fewer than 5. Continuous variables are expressed as medians with interquartile ranges (IQRs) and were compared using the Wilcox test. A two-sided *P*-value of less than 0.05 was considered statistically significant. OS, RFS, and LCSS were estimated using the Kaplan—Meier method. The Mantel—Cox log-rank test was used to compare survival between two groups. Univariate Cox proportional hazard analyses were used to evaluate the correlates of OS among patient clinicopathological characteristics and surgical methods. In multivariate Cox proportional hazard analyses, factors with a *P*-value less than 0.1 in univariate analyses and prognostic value in clinical practice were included. Subgroup analysis was performed in patients with different CTR. In this study, we included age, sex, tumor size, pTNM stage, histology, CTR, and MLND type in a logistic regression model to calculate the propensity scores between wedge resection and segmentectomy, and inverse probability was calculated from these scores. Inverse probability of treatment weighting (IPTW) analysis was performed based on this weight.

## Results

### Baseline characteristics

**Figure 1:**
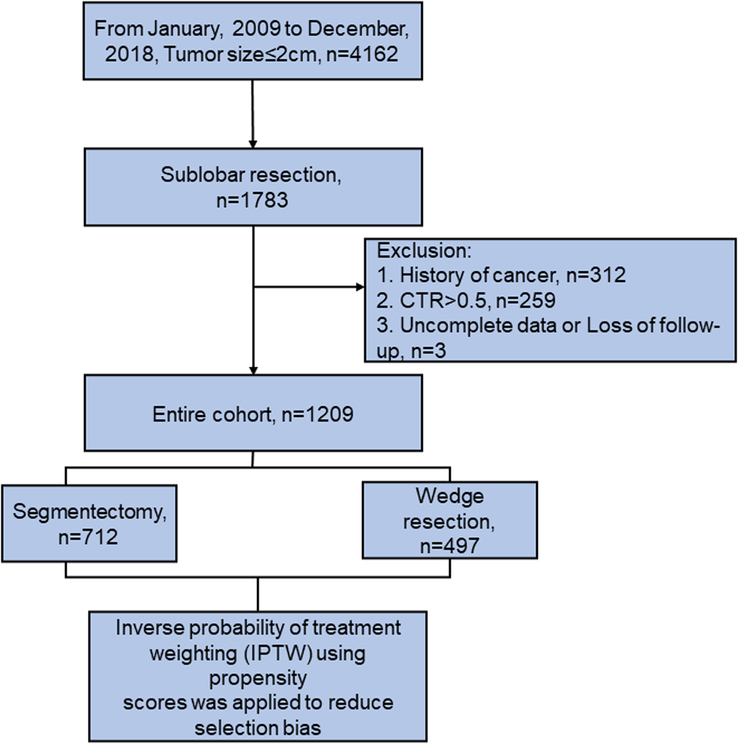
Patient flow diagram.

A total of 1209 patients who met our inclusion and exclusion criteria were screened out and analyzed, of which 497 (41.1%) and 712 (58.9%) patients underwent intentional wedge resection and segmentectomy, respectively (Fig. 1). Eighty-one (6.7%) patients were of advanced age (>70) and 825 (68.2%) patients were female in the entire cohort. All patients underwent VATS resection. Most patients (950, 78.6%) were radiologically noninvasive (CTR≤.25). All patients (except two patients with lung squamous cell carcinoma) had lung adenocarcinoma, and 550 (45.5%) patients had the invasive adenocarcinoma phenotype. Other baseline characteristics before and after matching are summarized in Table 1. Tumor size (*P*<0.001), pTNM stage (*P*=0.004), and LN resection (*P*<0.001) were significantly different between the two groups. After IPTW weighting, the distribution of those factors was balanced between the two groups (*P*>0.05).

**Table 1:** Patients baseline data in entire cohort and inverse probability of treatment weighting cohort.

	Entire cohort^a^			IPTW cohort^b^		
Variable	Segmentectomy *n*=712	Wedge resection *n*=497	*P*	Segmentectomy weights=605	Wedge resection weights=604	*P*
Age			0.962			0.944
>70	47 (6.6)	34 (6.8)		6.6	6.5	
≤70	665 (93.4)	463 (93.2)		93.4	93.5	
Sex			0.560			0.995
Male	221 (31.0)	163 (32.8)		31.8	31.8	
Female	491 (69.0)	334 (67.2)		68.2	68.2	
Symptom			0.927			0.723
Yes	123 (17.3)	84 (16.9)		17.1	17.9	
No	589 (82.7)	413 (83.1)		82.9	82.1	
CCI score			0.207			0.094
0	646 (90.7)	437 (87.9)		91	87.8	
1	62 (8.7)	54 (10.9)		8.5	10.9	
≥2	4 (0.6)	6 (1.2)		0.5	1.3	
Smoking			0.201			0.219
Yes	608 (85.4)	410 (82.5)		14.6	17.2	
No	104 (14.6)	87 (17.5)		85.4	82.8	
Location			0.449			0.353
Right	390 (54.8)	284 (57.1)		54.6	57.3	
Left	322 (45.2)	213 (42.9)		45.4	42.7	
Tumor (cm)			<0.001			0.994
>1	320 (44.9)	170 (34.2)		40.4	40.5	
≤1	392 (55.1)	327 (65.8)		59.6	59.5	
TNM Stage			0.004			1
0	64 (9.0)	62 (12.5)		10.5	10.5	
IA1	310 (43.5)	248 (49.9)		46.2	46.3	
IA2	238 (33.4)	125 (25.2)		30.0	30.1	
IB	100 (14.0)	62 (12.5)		13.3	13.2	
FEV1			0.857			0.625
>80	686 (96.3)	477 (96.0)		96.5	95.9	
≤80	26 (3.7)	20 (4.0)		3.5	4.1	
Multiprimary			0.283			0.385
Yes	62 (8.7)	34 (6.8)		8.3	6.9	
No	650 (91.3)	463 (93.2)		91.7	93.1	
Histology			0.106			1
AAH	14 (2.0)	17 (3.4)		2.6	2.6	
AIS	51 (7.2)	45 (9.1)		8	7.9	
MIA	302 (42.4)	228 (45.9)		43.8	43.8	
IA	344 (48.3)	206 (41.4)		45.5	45.5	
SQCC	1 (0.1)	1 (0.2)		0.1	0.1	
Systematic LN resection			<0.001			0.989
Yes	587 (82.4)	357 (71.8)		78.1	78.1	
No	125 (17.6)	140 (28.2)		21.9	21.9	
CTR			0.572			0.998
0–25	555 (77.9)	395 (79.5)		78.4	78.4	
25–50	157 (22.1)	102 (20.5)		21.6	21.6	

a Data are presented as number (percentage) of patients unless otherwise indicated.

b Data are presented as percentage of patients unless otherwise indicated.

AAH, atypical adenomatous hyperplasia; AIS, adenocarcinoma in situ; CCI, Charlson Comorbidity Index; CTR, consolidation-to-tumor ratio; LN, lymph node; MIA, minimally invasive adenocarcinoma; SQCC, Squamous cell carcinoma.

### Perioperative outcomes

In total, 74 (6.1%) patients had postoperative complications, and two patients underwent conversion from VATS to open surgery, resulting in a conversion rate of 0.2%. The reasons for conversion were hilar lymph node calcification and unexpected hemorrhage during dissection of severe pleural adhesions. There was no perioperative death. The detailed perioperative outcomes before and after matching are shown in Table 2. We noticed that wedge resection had significantly lower rates of postoperative complications than segmentectomy before (3.8 vs. 7.7%, *P*=0.008) and after matching (IPTW: 4 vs. 7.8%, *P*=0.007). Prolonged air leak (unweighted: *P*<0.001; IPTW: *P*<0.001) and pneumonia (unweighted: *P*=0.046; IPTW: *P*=0.03) were the main complications contributing to this difference. Moreover, operating time (unweighted: *P*<0.001; IPTW: *P*<0.001), length of postoperative hospital stay (unweighted: *P*<0.001; IPTW: *P*<0.001) and blood loss (unweighted: *P*<0.001; IPTW: *P*<0.001) were significantly lower in the wedge resection group.

**Table 2:** Short-term outcomes in entire cohort, and inverse probability of treatment weighting cohort.

	Entire cohort^a^			IPTW cohort^b^		
Variable	Segmentectomy	Wedge resection	*P*	Segmentectomy	Wedge resection	*P*
Complications^c^	55 (7.7)	19 (3.8)	0.008	7.8	4	0.007
Airleak (>5 days)	27 (3.8)	3 (0.6)	<0.001	3.8	0.8	<0.001
Chylothorax	6 (0.8)	2 (0.4)	0.482	0.8	0.5	0.725
Pneumonia	7 (1.0)	0 (0.0)	0.046	1	0	0.03
PE	1 (0.1)	0 (0.0)	1	0.1	0	1
Conversion	2 (0.3)	0 (0.0)	0.515	0.3	0	0.499
Operating time (median [IQR])	114.00 [95.00–140.00]	65.00 [54.00–85.00]	<0.001	110.00 [95.00–140.00]	65.00 [53.96–80.00]	<0.001
Postoperative stay (median [IQR])	4.00 [4.00–6.00]	3.00 [3.00–4.00]	<0.001	4.00 [4.00–6.00]	3.00 [3.00–4.00]	<0.001
Blood loss (median [IQR])	20.00 [20.00– 50.00]	20.00 [20.00–20.00]	<0.001	20.00 [20.00–50.00]	20.00 [20.00–20.00]	<0.001

a Data are presented as number (percentage) of patients unless otherwise indicated.

b Data are presented as percentage of patients unless otherwise indicated.

c Complications with lower fraction were not shown.

IPTW, inverse probability of treatment weighting; PE, pulmonary embolism.

### Survival outcomes

**Figure 2:**
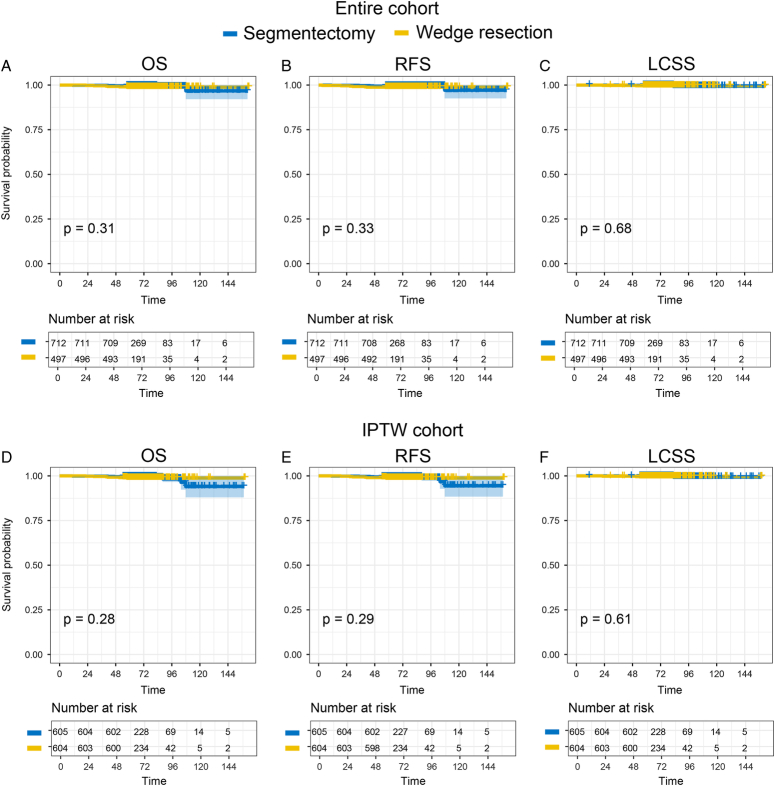
Long-term outcomes including overall survival (OS), recurrence-free survival (RFS), and lung cancer-specific survival (LCSS) in the entire cohort (A–C) and the inverse probability of treatment weighting (IPTW, D–F).

The median follow-up time was 70.1 (IQR, 62.6–82.7) months in the entire cohort. Thirteen patients died, and five patients had recurrence or metastasis. However, only four patients died because of lung cancer. The reasons for death in other patients were other primary cancers, cerebral infarctions, accidents, and viral pneumonia (Supplementary Table 1, Supplemental Digital Content 2, http://links.lww.com/JS9/C205). The 5-year OS rates were 98.8% in the wedge resection group and 99.6% in the segmentectomy group (*P*=0.31). The 5-year RFS rate was 98.8% in the wedge resection group and 99.4% in the segmentectomy group (*P*=0.33). The 5-year LCSS rate was 99.6% in the wedge resection group and 99.9% in the segmentectomy group (*P*=0.68, Supplementary Table 2, Supplemental Digital Content 2, http://links.lww.com/JS9/C205, Fig. 2A–C). The 10-year OS, RFS, and LCSS rates were 98.8, 98.8, and 99.6% for wedge resection and 96.7, 97.1, and 99.3% for segmentectomy, respectively. After weighting, all results were similar, and no significant prognostic differences were found between segmentectomy and wedge resection (IPTW: P_OS_=0.28, P_RFS_=0.29, P_LCSS_=0.61, Fig. 2D–F).

Through univariable Cox analysis, we found that advanced age, CCI score, tumor size, and pTNM stage were significant factors for OS and RFS, and smoking history was a significant factor for LCSS (Supplementary Table 3, Supplemental Digital Content 3, http://links.lww.com/JS9/C205). Multivariable Cox analysis (Table 3) showed that only advanced age was a significant factor for OS (HR=8.54, 95% CI: 2.44–29.91, *P*<0.001) and RFS (HR=8.34, 95% CI: 2.39–29.02, *P*<0.001). There were no significant differences between segmentectomy and wedge resection in OS (HR=1.98, 95% CI: 0.59–6.68, *P*=0.270) or RFS (HR=1.88, 95% CI: 0.56–6.31, *P*=0.307).

**Table 3:** Multivariable cox proportional analysis for OS, RFS, and LCSS.

	OS			RFS			LCSS		
	HR	95% CI	*P*	HR	95% CI	*P*	HR	95% CI	*P*
Age (>70 vs ≤70)	8.54	2.44–29.91	0.001	8.34	2.39–29.02	0.001	5.06	0.48–52.75	0.176
Sex (Male vs Female)	1.48	0.3–7.24	0.627	1.42	0.29–6.91	0.668	0.86	0.03–29.81	0.935
Group (Wedge vs Seg)	1.98	0.59–6.68	0.27	1.88	0.56–6.31	0.307	1.76	0.24–13.15	0.581
CCI score (≥1 vs 0)	0.74	0.15–3.7	0.716	0.78	0.16–3.89	0.763	0	0-Inf^a^	0.999
Smoking (Yes vs No)	2.56	0.48–13.54	0.269	2.38	0.45–12.53	0.306	19.42	0.54–698.25	0.105
Tumor (>1 vs ≤1)	4.47	0.91–22.03	0.066	4.48	0.91–22.07	0.066	2.88	0.27–30.21	0.378
CTR(>0.25 vs ≤0.25)	1.19	0.34–4.2	0.783	1.21	0.34–4.26	0.767	2.54	0.34–19.2	0.367

a No LCSS events in CCI score ≥1 group.

CCI, Charlson Comorbidity Index; CTR, consolidation-to-tumor ratio; HR, hazard ratio; LCSS, lung cancer-specific survival; OS, overall survival; RFS, recurrence-free survival.

### Subgroup analysis

**Figure 3:**
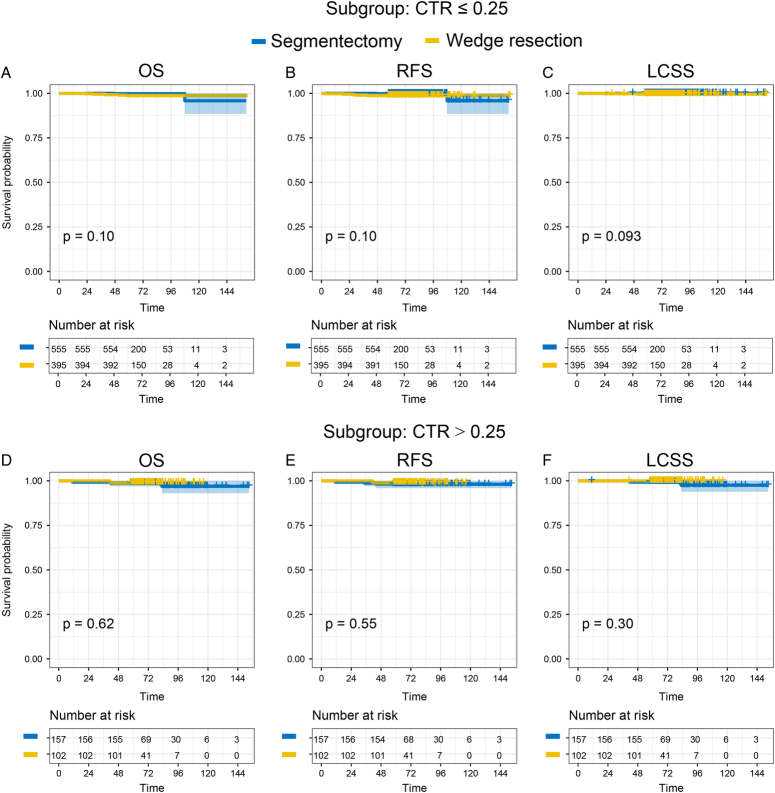
Subgroup analysis of survival outcomes in patients with different CTR. (A–C) overall survival (OS), recurrence-free survival (RFS), and lung cancer-specific survival (LCSS) in patients with CTR ≤0.25. (D-F) OS, RFS, and LCSS in patients with CTR >0.25.

We conducted a subgroup analysis of patients with a CTR ≤0.25 or a CTR >0.25. In the CTR >0.25 subgroup, the 5-year OS, RFS, and LCSS rates were 99.6, 99.4, and 99.9% for patients who underwent segmentectomy; the 5-year OS, RFS, and LCSS rates were 98.8, 98.8, and 99.6% for patients who underwent wedge resection. The survival curves showed that there were no significant differences between the two surgical approaches in terms of a CTR ≤0.25 (P_OS_=0.10, P_RFS_=0.10, P_LCSS_=0.093, Fig. 3A–C) or a CTR >0.25 (P_OS_=0.62, P_RFS_=0.55, P_LCSS_=0.30, Fig. 3D–F).

## Discussion

In this study, we demonstrated that for patients with tumor sizes less than 2 cm and CTRs less than 0.5, wedge resection has long-term survival outcomes, including OS, RFS, and LCSS rates, that are comparable to segmentectomy. The perioperative outcomes of wedge resection were much better. The IPTW analysis revealed that all results were robust after balancing clinical characteristics between the two groups.

This study confirmed that wedge resection is one of the safest procedures for treating lung cancer. Wedge resection had an operation time that was almost half the time of segmentectomy and the recovery time that was one day less. Only 3.8% of patients who underwent wedge resection experienced complications, which is half the complication rate in patients who underwent segmentectomy (7.7%). Compared to other studies, such as CALGB140503, JCOG0802, and JCOG0804, which reported complication rates ranging from 14 to 37%^11,25,26^, the present study had a much lower complication rate and no perioperative death. The occurrence of postoperative complications depends on many factors. For example, segmentectomy requires surgeons to perform approximately 80-100 procedures to master the learning curve^27,28^. On the other hand, anatomical variety and location influence the difficulty of segmentectomy. Therefore, when making a decision, surgeons should take into account the tumor’s status as well as surgeons’ capabilities.

The post-hoc analysis of the RCT (CALGB140503) found that there was no clinically meaningful difference in DFS and OS between patients treated by wedge resection and segmentectomy^14^. However, there was no detailed information about the CTR of the tumor, which was directly related to the invasiveness and prognosis^9^. According to previous reports, patients with a tumor size ≤2 cm and a CTR ≤0.5 had excellent survival outcomes. However, the optimal sublobar resection procedure (segmentectomy or wedge resection) for those patients remains debatable^16–18^. Consistent with previous reports, patients with a tumor size ≤2 cm and a CTR ≤0.5 had excellent survival outcomes. In this study, the 5-year OS rate was 98.8% in wedge resection and 99.6% in segmentectomy, and the 10-year OS rates were 98.8 and 96.7%, respectively. JCOG0201 determined that a CTR ≤0.25 was a radiologically noninvasive lesion, and its long-term follow-up results showed that the 10-year OS rate was 94.0%. For those patients, the 5-year RFS rate was 99.7% in the JCOG0804 study, with no local recurrence^11^, and the 10-year RFS rate was 98.6%^29^. JCOG1211 found that for patients with tumor sizes <3 cm and a CTR ranging from 0.25 to 0.5 who underwent segmentectomy, the 5-year RFS rate was 98.0%^12^. We found that the potential risk factors were advanced age and tumor size, which were significant in the univariable analysis. In the multivariable analysis, advanced age was a significant risk factor for OS and RFS but not for LCSS. Aging-related diseases, including cardiovascular disease, neurodegeneration, and metabolic disease, are as dangerous as malignancy. GGO-dominant patients had good survival outcomes; thus, in this study, CTR was not significant in our Cox proportional model. The outcomes of the CTR subgroup analyses were robust. For patients with a CTR between 0.25 and 0.5, the 5-year OS rates were 98.8 and 99.6% for wedge resection and segmentectomy, respectively. Our results were consistent with a recent report^30^; however, the follow-up time in our study was almost twice as long as this study. To the best of our knowledge, we are the first study to report the long-term survival outcomes of the two sublobar resection approaches in the largest sample of patients with the longest follow-up time. We hope that these results will fill the gaps in existing research.

Although those patients have excellent survival after resection, sufficient resection margins should not be ignored during surgery^31^. According to the National Comprehensive Cancer Network (NCCN), an adequate surgical margin is defined as a surgical margin distance of ≥2 cm or at least the total tumor size. Akamine and colleagues reported that the probability of obtaining adequate surgical margins was significantly higher with segmentectomy (71.4%) versus wedge resection (59.5%)^17^. One study reported that the surgical margin was not a significant risk factor for GGO-dominant patients^32^, but the evidence level is still limited. In this study, we adhered to the surgical margin recommended by the NCCN. Moreover, an important step in the surgical therapy of lung cancer is the examination of the lymph nodes. Theoretically, only the lymph nodes in the hilum and mediastinum can be evaluated by wedge resection, while intrapulmonary lymph nodes cannot be evaluated. However, no patients in the wedge resection group had lymph node recurrence during the follow-up period. Instead, there was one case of lymph node recurrence in the segmentectomy group; however, this patient had multiple systemic metastases, which means that this patient may have a more aggressive tumor. Recently, a multicenter prospective clinical trial revealed that none of the GGO-dominant patients had lymph node metastasis^33^. As a result, intrapulmonary lymph node examination is not always required for this group of patients, and lobe-specific evaluation is sufficient. Hattori *et al*.^34^ has found that for multiple primary lung cancers, multifocal GGOs did not compromise survivals. In this study^34^, the 5-year OS in multifocal GGOs was 97.2%, which is consistent with our study, the 5-year OS in solid nodule with additional GGO patients was 82.1%; and the 5-year OS in solid nodule with additional solid nodule was 41.3%. Moreover, Chen *et al*.^35^ reported that sublobar resection is acceptable for patients with MPLC at an early-stage. And in our Cox proportional model, presence multiple primary GGOs was not significantly associated with survival (OS: HR=1.33, 95% CI: 0.17–10.48, *P*=0.787; RFS: HR=1.26, 95% CI: 0.16–9.88, *P*=0.828). Thus, we think GGO patients with multiple primaries has similar survival outcomes compared to those without multiple primaries and suitable to sublobar resection.

This study is a retrospective study in a single center. Although our median follow-up period was 70.1 months, a longer follow-up may be required to validate the study’s findings. To avoid the limitations of retrospective studies and obtain a more convincing result, we conducted a prospective clinical trial to compare the long-term survival outcomes between segmentectomy and wedge resection for those patients (NCT02718365), in which enrollment of patients had been completed. We are still eagerly waiting to report the results of the long-term follow-ups. However, it remains to be explored whether these findings can be applied to non-Asian populations.

## Conclusion

In comparison to segmentectomy, intentional wedge resection offers improved perioperative outcomes and comparable long-term outcomes for GGO-dominant patients with tumor sizes less than 2 cm. Importantly, our study provides new evidence that wedge resection could achieve satisfactory outcomes for patients with CTR between 0.25 and 0.5. Future international, multicenter studies may still be warranted to further prove this conclusion in real-world clinical practice.

## Ethical approval

This study was approved by the Institutional Review Board (IRB) of West China Hospital (no. 2024-56).

## Consent

No personal information’s were mentioned in this article. Informed consent was waived for this research.

## Source of funding

This research was supported by the 1.3.5 Project for Disciplines of Excellence, West China Hospital, Sichuan University (No. ZYJC21002 to Lunxu Liu) National Key R&D Program of China (No 2021YFC2500905 to Chengwu Liu); the Science and Technology Project of the Health Commission of Sichuan Province, China (No. 21PJ008 to Liang Xia); Key Projects of Sichuan Province (No. 2022YFS0208 to Liang Xia).

## Author contribution

L.L.X., L.C.W., and Y.Z.Y.: designed the study; Y.Z.Y., L.Y.M., G.C.L., X.L., Z.W.H., X.C.J., M.J.D., L.H., Z.Y.K., L.F., M.L., and P.Q.: collected the data; Y.Z.Y., L.C.W., and L.Y.M.: analyzed the data. All authors had full access to the data, verified the underlying data, and contributed to data interpretation and the review, revision, and approval of the report.

## Conflicts of interest disclosures

All authors declare that there are no conflicts of interest.

## Research registration unique identifying number (UIN)

1. Name of the registry: Chinese Clinical Trial Registry.
2. Unique identifying number or registration ID: ChiCTR2400079887.
3. Hyperlink to your specific registration (must be publicly accessible and will be checked): https://www.chictr.org.cn/hvshowproject.html?id=244571.

## Guarantor

Lunxu Liu.

## Data availability statement

All relevant data are within the paper. The raw data are available from the corresponding author on reasonable request.

## Provenance and peer review

Not commissioned, externally peer-reviewed.

## Assistance with the study

None.

## Presentation

None.

## Study design

Cohort study.

## Supplementary Material

SUPPLEMENTARY MATERIAL
