# Supplementary material for: Intentional wedge resection versus segmentectomy for ≤2 cm ground-glass-opacity-dominant non-small cell lung cancer: a real-world study using inverse probability of treatment weighting
Source: Int J Surg. 2024 Mar 21;110(7):4231–9. doi: 10.1097/JS9.0000000000001361 (PMC11254288; doi:10.1097/JS9.0000000000001361)
Supplement: SUPPLEMENTARY MATERIAL [file js9-110-4231-s002.docx]

**Table 1 Patients baseline data in entire cohort and inverse probability of treatment weighting cohort.**

| Variable | Entire cohort ^a^ | |  |  | IPTW cohort^b^ | |  |
| --- | --- | --- | --- | --- | --- | --- | --- |
|  | Segmentectomy  n=712 | Wedge resection  n=497 | P-value |  | Segmentectomy  weights=605 | Wedge resection  weights=604 | P-value |
| Age |  |  | 0.962 |  |  |  | 0.944 |
| >70 | 47 (6.6) | 34 (6.8) |  |  | 6.6 | 6.5 |  |
| ≤70 | 665 (93.4) | 463 (93.2) |  |  | 93.4 | 93.5 |  |
| Sex |  |  | 0.560 |  |  |  | 0.995 |
| Male | 221 (31.0) | 163 (32.8) |  |  | 31.8 | 31.8 |  |
| Female | 491 (69.0) | 334 (67.2) |  |  | 68.2 | 68.2 |  |
| Symptom |  |  | 0.927 |  |  |  | 0.723 |
| Yes | 123 (17.3) | 84 (16.9) |  |  | 17.1 | 17.9 |  |
| No | 589 (82.7) | 413 (83.1) |  |  | 82.9 | 82.1 |  |
| CCI score |  |  | 0.207 |  |  |  | 0.094 |
| 0 | 646 (90.7) | 437 (87.9) |  |  | 91 | 87.8 |  |
| 1 | 62 (8.7) | 54 (10.9) |  |  | 8.5 | 10.9 |  |
| ≥2 | 4 (0.6) | 6 (1.2) |  |  | 0.5 | 1.3 |  |
| Smoking |  |  | 0.201 |  |  |  | 0.219 |
| Yes | 608 (85.4) | 410 (82.5) |  |  | 14.6 | 17.2 |  |
| No | 104 (14.6) | 87 (17.5) |  |  | 85.4 | 82.8 |  |
| Location |  |  | 0.449 |  |  |  | 0.353 |
| Right | 390 (54.8) | 284 (57.1) |  |  | 54.6 | 57.3 |  |
| Left | 322 (45.2) | 213 (42.9) |  |  | 45.4 | 42.7 |  |
| Tumor (cm) |  |  | <0.001 |  |  |  | 0.994 |
| >1 | 320 (44.9) | 170 (34.2) |  |  | 40.4 | 40.5 |  |
| ≤1 | 392 (55.1) | 327 (65.8) |  |  | 59.6 | 59.5 |  |
| TNM Stage |  |  | 0.004 |  |  |  | 1 |
| 0 | 64 (9.0) | 62 (12.5) |  |  | 10.5 | 10.5 |  |
| IA1 | 310 (43.5) | 248 (49.9) |  |  | 46.2 | 46.3 |  |
| IA2 | 238 (33.4) | 125 (25.2) |  |  | 30.0 | 30.1 |  |
| IB | 100 (14.0) | 62 (12.5) |  |  | 13.3 | 13.2 |  |
| FEV1( |  |  | 0.857 |  |  |  | 0.625 |
| >80 | 686 (96.3) | 477 (96.0) |  |  | 96.5 | 95.9 |  |
| ≤80 | 26 (3.7) | 20 (4.0) |  |  | 3.5 | 4.1 |  |
| Multi-Primary |  |  | 0.283 |  |  |  | 0.385 |
| Yes | 62 (8.7) | 34 (6.8) |  |  | 8.3 | 6.9 |  |
| No | 650 (91.3) | 463 (93.2) |  |  | 91.7 | 93.1 |  |
| Histology |  |  | 0.106 |  |  |  | 1 |
| AAH | 14 (2.0) | 17 (3.4) |  |  | 2.6 | 2.6 |  |
| AIS | 51 (7.2) | 45 (9.1) |  |  | 8 | 7.9 |  |
| MIA | 302 (42.4) | 228 (45.9) |  |  | 43.8 | 43.8 |  |
| IA | 344 (48.3) | 206 (41.4) |  |  | 45.5 | 45.5 |  |
| SQCC | 1 (0.1) | 1 (0.2) |  |  | 0.1 | 0.1 |  |
| Systematic LN resection |  |  | <0.001 |  |  |  | 0.989 |
| Yes | 587 (82.4) | 357 (71.8) |  |  | 78.1 | 78.1 |  |
| No | 125 (17.6) | 140 (28.2) |  |  | 21.9 | 21.9 |  |
| CTR |  |  | 0.572 |  |  |  | 0.998 |
| 0~25 | 555 (77.9) | 395 (79.5) |  |  | 78.4 | 78.4 |  |
| 25~50 | 157 (22.1) | 102 (20.5) |  |  | 21.6 | 21.6 |  |

a Data are presented as number(percentage) of patients unless otherwise indicated.

b Data are presented as percentage of patients unless otherwise indicated.

Abbreviations: CCI: Charlson Comorbidity Index; AAH: atypical adenomatous hyperplasia; MIA: minimally invasive adenocarcinoma; AIS: adenocarcinoma in situ; SQCC: Squamous cell carcinoma; LN: lymph node; CTR: consolidation-to-tumor ratio

**Table 2. Short-term outcomes in entire cohort, and inverse probability of treatment weighting cohort**.

| Variable | Entire cohort^a^ |  |  |  | IPTW cohort^b^ |  |  |
| --- | --- | --- | --- | --- | --- | --- | --- |
|  | Segmentectomy | Wedge resection | P-value |  | Segmentectomy | Wedge resection | P-value |
| Complications^c^ | 55 (7.7) | 19 (3.8) | 0.008 |  | 7.8 | 4 | 0.007 |
| Airleak(>5d) | 27 (3.8) | 3 (0.6) | <0.001 |  | 3.8 | 0.8 | <0.001 |
| Chylothorax | 6 (0.8) | 2 (0.4) | 0.482 |  | 0.8 | 0.5 | 0.725 |
| Pneumonia | 7 (1.0) | 0 (0.0) | 0.046 |  | 1 | 0 | 0.03 |
| PE | 1(0.1) | 0(0.0) | 1 |  | 0.1 | 0 | 1 |
| Conversion | 2(0.3) | 0(0.0) | 0.515 |  | 0.3 | 0 | 0.499 |
| Operating time  (median[IQR]) | 114.00[95.00,140.00] | 65.00[54.00,85.00] | <0.001 |  | 110.00[95.00, 140.00] | 65.00[53.96,80.00] | <0.001 |
| Postoperative stay  (median[IQR]) | 4.00 [4.00, 6.00] | 3.00[3.00, 4.00] | <0.001 |  | 4.00[4.00, 6.00] | 3.00[3.00,4.00] | <0.001 |
| Blood loss  (median[IQR]) | 20.00[20.00, 50.00] | 20.00[20.00, 20.00] | <0.001 |  | 20.00[20.00, 50.00] | 20.00[20.00, 20.00] | <0.001 |

a Data are presented as number(percentage) of patients unless otherwise indicated.

b Data are presented as percentage of patients unless otherwise indicated.

c. Complications with lower fraction were not shown.

Abbreviations: IPTW: inverse probability of treatment weighting; PE: pulmonary embolism

**Table3** **Multivariable cox proportional analysis for OS, RFS, LCSS**

|  | OS |  |  |  | RFS |  |  |  | LCSS |  |  |
| --- | --- | --- | --- | --- | --- | --- | --- | --- | --- | --- | --- |
|  | HR | 95%CI | P |  | HR | 95%CI | P |  | HR | 95%CI | P |
| Age (>70 vs ≤70) | 8.54 | 2.44-29.91 | 0.001 |  | 8.34 | 2.39-29.02 | 0.001 |  | 5.06 | 0.48-52.75 | 0.176 |
| Sex (Male vs Female) | 1.48 | 0.3-7.24 | 0.627 |  | 1.42 | 0.29-6.91 | 0.668 |  | 0.86 | 0.03-29.81 | 0.935 |
| Group (Wedge vs Seg) | 1.98 | 0.59-6.68 | 0.27 |  | 1.88 | 0.56-6.31 | 0.307 |  | 1.76 | 0.24-13.15 | 0.581 |
| CCI score (≥1 vs 0) | 0.74 | 0.15-3.7 | 0.716 |  | 0.78 | 0.16-3.89 | 0.763 |  | 0 | 0-Inf^a^ | 0.999 |
| Smoking (Yes vs No) | 2.56 | 0.48-13.54 | 0.269 |  | 2.38 | 0.45-12.53 | 0.306 |  | 19.42 | 0.54-698.25 | 0.105 |
| Tumor (>1 vs ≤1) | 4.47 | 0.91-22.03 | 0.066 |  | 4.48 | 0.91-22.07 | 0.066 |  | 2.88 | 0.27-30.21 | 0.378 |
| CTR(>0.25 vs ≤0.25) | 1.19 | 0.34-4.2 | 0.783 |  | 1.21 | 0.34-4.26 | 0.767 |  | 2.54 | 0.34-19.2 | 0.367 |

a No LCSS events in CCI score≥1 group.

Abbreviations: OS: overall survival; RFS: recurrence-free survival; LCSS: lung cancer specific survival; HR: hazard ratio; CI: confidence interval; CCI: Charlson Comorbidity Index; CTR: consolidation-to-tumor ratio

**Supplementary Table 1. Death or recurrence of wedge resection and segmentectomy groups**

| Group | OS | Death reason | RFS | Recurrence or metastasis site |
| --- | --- | --- | --- | --- |
| Segmentectomy | 82.9 | Lung cancer | 43.9 | Brain, bone and mediastinum LN |
|  | 107.9 | Cerebrovascular accident | 107.9 | Lung |
|  | 46.9 | Cutaneous T cell lymphoma | 46.9 | - |
|  | 41.4 | Lung cancer | 34.7 | Bone, lung |
|  | 11.0 | Cerebrovascular accident | 11 |  |
|  | 103.8 | Virus pneumonia | 103.8 |  |
|  | 88.1 | accident | 88.1 |  |
| Wedge Resection | 29.1 | Accident | 29.1 |  |
|  | 51.0 | Lung cancer | 26.6 | Bone |
|  | 41.0 | Esophageal cancer | 41.0 |  |
|  | 57.7 | Virus pneumonia | 57.7 |  |
|  | 39.5 | Pancreatic cancer | 39.5 |  |
|  | 22.3 | Lung cancer | 18.9 | Bone, adrenal gland |

**Supplementary Table 2. Survival outcomes in wedge resection and segmentectomy group.**

|  |  | 5-year |  |  |  |  |  | 10-year |  |  |  |  |  |  |
| --- | --- | --- | --- | --- | --- | --- | --- | --- | --- | --- | --- | --- | --- | --- |
|  |  | OS | 95%CI | RFS | 95%CI | LCSS | 95%CI | OS | 95%CI |  | RFS | 95%CI | LCSS | 95%CI |
| Entire | Segmentectomy | 99.6 | 99.1-100 | 99.4 | 98.9-100 | 99.9 | 99.6-100 | 96.7 | 92.1-100 |  | 97.1 | 92.6-100 | 99.3 | 98.3-100 |
|  | Wedge Resection | 98.8 | 97.8-99.8 | 98.8 | 97.8-99.8 | 99.6 | 99.0-100 | 98.8 | 97.8-99.8 |  | 98.8 | 97.8-99.8 | 99.6 | 99.0-100 |
| IPTW | Segmentectomy | 99.6 | 99.1-100 | 99.5 | 99.0-100 | 99.9 | 99.6-100 | 97.1 | 93.1-100 |  | 97.4 | 93.4-100 | 99.4 | 98.5-100 |
|  | Wedge Resection | 98.8 | 97.9-99.8 | 98.8 | 97.9-99.8 | 99.6 | 99.0-100 | 98.8 | 97.9-99.8 |  | 98.8 | 97.9-99.8 | 99.6 | 99.0-100 |

**Supplementary Table 3. Univariable cox proportional analysis for OS, RFS, LCSS**

|  | OS |  |  |  | RFS |  |  |  | LCSS |  |  |
| --- | --- | --- | --- | --- | --- | --- | --- | --- | --- | --- | --- |
|  | HR | 95%CI | P |  | HR | 95%CI | P |  | HR | 95%CI | P |
| Age (>70 vs ≤70) | 11.05 | 3.36-36.31 | <0.001 |  | 11.45 | 3.48-37.6 | <0.001 |  | 4.36 | 0.45-42.11 | 0.203 |
| Sex (Male vs Female) | 2.59 | 0.79-8.48 | 0.117 |  | 2.56 | 0.78-8.39 | 0.273 |  | 6.65 | 0.69-63.96 | 0.101 |
| Group (Wedge vs Seg) | 1.83 | 0.56-6.01 | 0.32 |  | 1.8 | 0.55-5.9 | 0.334 |  | 1.52 | 0.21-10.79 | 0.677 |
| Symptom (Yes vs No) | 0.87 | 0.18-4.12 | 0.862 |  | 0.94 | 0.2-4.43 | 0.935 |  | 0.75 | 0.08-7.4 | 0.808 |
| CCI score (≥1 vs 0) | 1.83 | 0.39-8.5 | 0.442 |  | 1.96 | 0.42-9.08 | 0.390 |  | 0^a^ | 0-Inf ^a^ | 0.999 ^a^ |
| Smoking (Yes vs No) | 3.18 | 0.93-10.89 | 0.065 |  | 3.12 | 0.91-10.67 | 0.069 |  | 17.01 | 1.77-163.82 | 0.014 |
| Location (Right vs Left) | 0.63 | 0.19-2.08 | 0.453 |  | 0.64 | 0.2-2.1 | 0.463 |  | 0.77 | 0.11-5.5 | 0.798 |
| Tumor (>1 vs ≤1) | 6.22 | 1.34-28.88 | 0.02 |  | 6.37 | 1.37-29.55 | 0.018 |  | 4.18 | 0.43-40.26 | 0.216 |
| Stage |  |  |  |  |  |  |  |  |  |  |  |
| IA1 | Ref |  |  |  | Ref |  |  |  | Ref |  |  |
| IA2 | 5.09 | 1.06-24.56 | 0.043 |  | 5.22 | 1.08-25.17 | 0.039 |  | 2.90 | 0.26-32.1 | 0.385 |
| IB | 3.07 | 0.43-21.92 | 0.998 |  | 3.24 | 0.45-25.17 | 0.241 |  | 3.02 | 0.19-48.72 | 0.999 |
| Multi-Primary (Yes vs No) | 1.33 | 0.17-10.48 | 0.787 |  | 1.26 | 0.16-9.88 | 0.828 |  | 0 ^a^ | 0-Inf ^a^ | 0.999 ^a^ |
| Histology (IA vs other) | 1.69 | 0.48-5.99 | 0.418 |  | 1.83 | 0.52-6.44 | 0.348 |  | 622975105.55 ^a^ | 0-Inf ^a^ | 0.999 ^a^ |
| LN (selective vs systematic) | 0.77 | 0.2-2.9 | 0.698 |  | 0.77 | 0.21-2.93 | 0.707 |  | 0.82 | 0.09-7.88 | 0.863 |
| CTR(25~50 vs 0~25) | 1.92 | 0.56-6.61 | 0.299 |  | 1.97 | 0.57-6.78 | 0.281 |  | 3.45 | 0.48-24.57 | 0.216 |

a No LCSS events in patients with CCI score >1 or multiple primary or non-invasive adenocarcinoma group.
